# Extracting Teeth

**Published:** 1896-02

**Authors:** D. Siddall

**Affiliations:** The Dalles, Oregon.


ARTICLE VI.

### EXTRACTING TEETH.

BY DR. D. SIDDALL, THE DALLES, OREGON.

Read before the Oregon State Dental Association at Astoria, August 14, 1895.

When I accepted the invitation to write upon this subject I did not realize the gravity of the question, and feel now that I am not adequate to the task, it being, as I believe, the balance sheet of all dentistry,—extracting being the most important operation of a surgical nature falling upon the care of the dentist, and coming as it does when the tooth cannot be saved by filling or by any other means known to the profession.

In order to extract any tooth successfully, it is necessary to know what its configuration normally ought to be; also the proper instrument to be used. The condition to which decay has reduced the tooth must never be lost sight of. In all cases I remove the gum with the lance, as I do not believe in pushing it away with the forceps, nor take the chance of stripping it off the jaw by means of the pulling process, as every portion of the gum should be left to form, as it were, a cushion over the alveolar or bone formation; and in many cases I let my lance glide down outside the alveolar cavity in cases of friable teeth to the depth of one-eighth of an inch or more, in order that the forceps may pass down and bite off a portion of the process, and thus firmly grasp a portion of the fang. I then detach the tooth from the walls of its socket in the direction where least resistance is likely to be met. In this way you may remove a fang, or even a tooth with three roots, with but very little trouble or pain to your patient, even in case the fangs may be malformed, or bent, or curved, in such a manner as to be difficult or altogether impossible to extract by any ordinary means.

Again, there are other styles of teeth which are extremely difficult to extract—such as the wisdom teeth and lower molars, when broken off very low down; as the jawbone of these teeth are frequently found to extend out from the tooth almost at right angles and is very thick, thereby resisting the action of the forceps or any other instrument I have ever been able to procure in the market. These teeth I seldom attempt to remove by the pulling process, but simply roll them out by the use of my improved dental elevators, which consists of four instruments, two right and two left, two of them being large ones and two small ones, which I use in the following manner: After removing the gum with the lance, as above described (which you will find very necessary around the wisdom teeth), I insert the small elevator into the handle, which stands at right angles, grasp the handle in the right hand with three fingers and the thumb, extending the fore, or index-finger to the end and on top of the elevator, so as to guide the instrument and prevent it from slipping; pass the blade or beak of the instrument down alongside the root I wish to extract, with the flat side facing it, and the convex surface next the adjoining tooth or root, if there be one; but, if no tooth or root is near the one to be extracted, I then press the elevator down between the jaw bone and the root sufficiently deep, then, by turning it sideways, the teeth of the instrument will engage the root and it will be easily raised from its place. Then, if another root remains alongside from where the first one was removed, place the large elevator in the handle and grasp same as before and drop the beak or lip into the socket from whence the first root was taken, and, by turning it sideways, as in the first instance, it will bite off the thin bit of process alongside the root, when the teeth of the instrument will engage the root, and it, too, will be lifted out of its place without the least difficulty.

The upper wisdom teeth, as a rule, can be removed intact by one motion of the small instrument, but in some cases I use the large elevator if I find the roots stand too far back from the molar to be reached by the small one. You will please bear in mind that the wisdom teeth are very often found to stand obliquely to the molars, and are easily rolled out, if you can only reach them low enough down; hence, the benefit of the elevator, as you can place it where you please and always get the offender, when that noble instrument, the physic forceps, fails.

Now, in conclusion, I wish to say a word to the young men of this our grand profession, which is second to none, inasmuch as we relieve and prevent more suffering than all the physicians in the world. Let us imagine a person coming into your office, who is a poor suffering wreck of humanity. They may have had a tooth broken off and roughly handled by some inexperienced hand, and they wish you to take out a very, very bad tooth, as they are most apt to say; and they don't want you to hurt them, at the same time inform you that they don't want to take an anesthetic, as their physician has told them that they have heart disease. Now, what is the first thing to be done in this case? I will tell you what I do. I never attempt to even look in their mouth until I have first gained their confidence, and this I never fail to do.

I tell them that I shall treat them gently, kindly, and yet thoroughly, so that when I take hold of the tooth it is sure to come out; I also make them believe that by means of a local application I can to a great extent relieve the pain. This I do in most cases by a liberal use of equal parts of creosote and tincture of iodine on the gums around the tooth which simply stops circulation for the time being. But they may say: "Oh, my tooth has stopped aching now, so I think I will go home." "Yes," I will say, "what is that? Simply the action of your mind over the physical system. Now, if your mind can stop the worst kind of pain, why won't it make me hurt you awfully, if you only think so?" Now, gentlemen, while I do not exactly believe in hypnotism, I do believe in working on the mind; it is the principal thing with many you come in contact with. Try it, and by dexterously using your instruments your patient will get out of your chair, go away laughing and send you all their friends they can, to be hurt in the same way.—*Pacific Stomatological Gazette.*

